# Supplementary material for: Comparison of circulating tumor cell (CTC) detection rates with epithelial cell adhesion molecule (EpCAM) and cell surface vimentin (CSV) antibodies in different solid tumors: a retrospective study
Source: PeerJ. 2021 Mar 2;9:e10777. doi: 10.7717/peerj.10777 (PMC7934682; doi:10.7717/peerj.10777)
Supplement: Supplemental Information 2 — Comparison of CTC count with EpCAM and CSV antibodies in the same patients. LC = lung cancer; CRC = colorectal cancer; BCa = breast cancer; GC = gastric cancer; PDAC = pancreatic ductal adenocarcinoma; CC = cervical cancer; EC = esophageal cancer; HCC = hepatocellular carcinoma; OC = ovarian cancer. [file peerj-09-10777-s002.pdf]

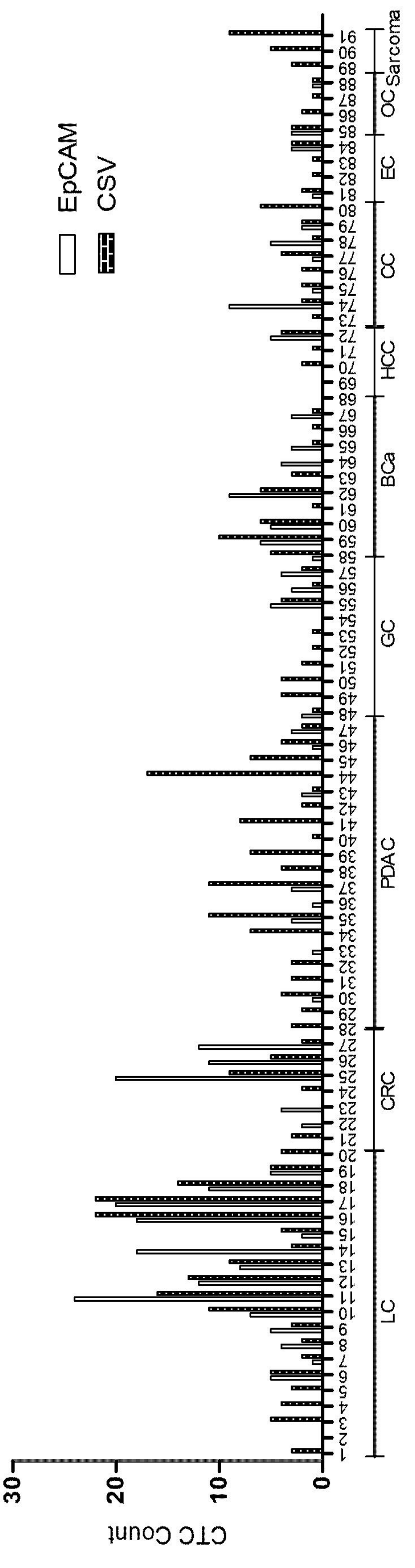

Supplement Figure 1

Comparison of CTC count with EpCAM and CSV antibodies in the same patients. LC = lung cancer; CRC = colorectal cancer; BCa = breast cancer; GC = gastric cancer; PDAC = pancreatic ductal adenocarcinoma; CC = cervical cancer; EC = esophageal cancer; HCC = hepatocellular carcinoma; OC = ovarian cancer.
