# Supplementary material for: Comparison of circulating tumor cell (CTC) detection rates with epithelial cell adhesion molecule (EpCAM) and cell surface vimentin (CSV) antibodies in different solid tumors: a retrospective study
Source: PeerJ. 2021 Mar 2;9:e10777. doi: 10.7717/peerj.10777 (PMC7934682; doi:10.7717/peerj.10777)
Supplement: Supplemental Information 3 [file peerj-09-10777-s003.pdf]

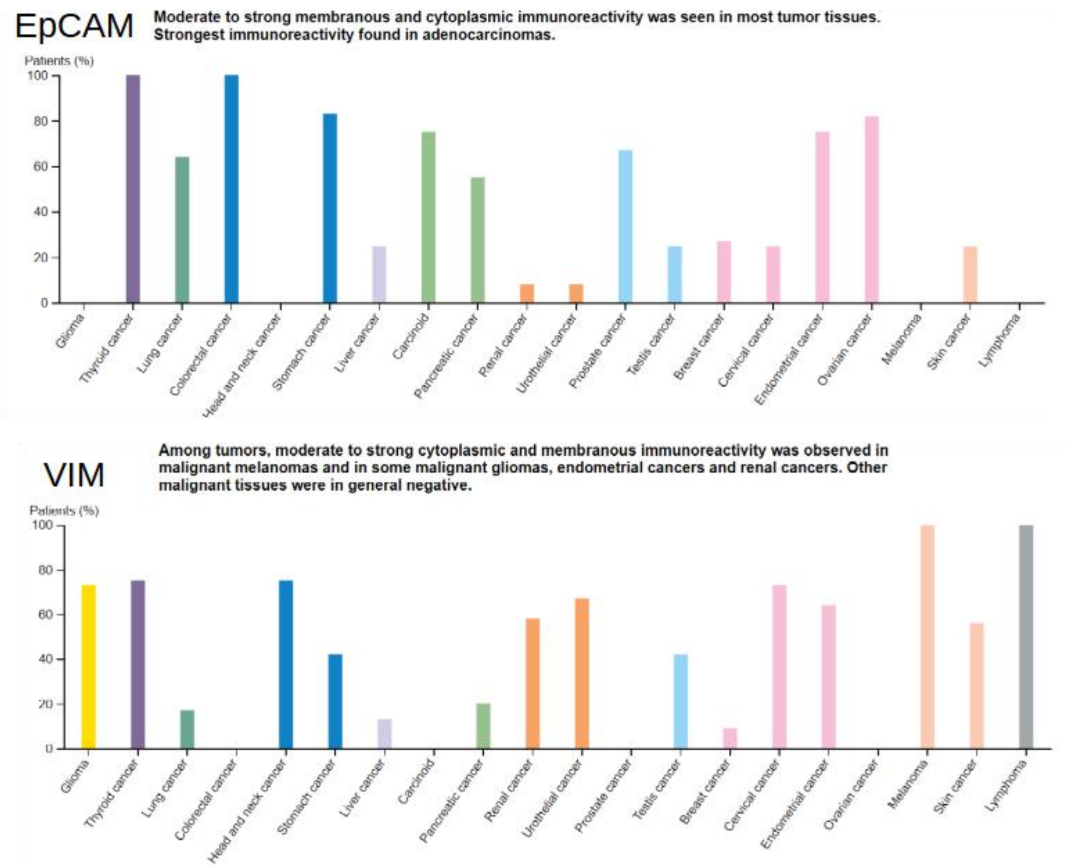

Supplement Figure 2

The Cancer Genome Atlas (TCGA) dataset of EpCAM and Vim expression in different cancers.
