# Supplementary material for: Comparison of circulating tumor cell (CTC) detection rates with epithelial cell adhesion molecule (EpCAM) and cell surface vimentin (CSV) antibodies in different solid tumors: a retrospective study
Source: PeerJ. 2021 Mar 2;9:e10777. doi: 10.7717/peerj.10777 (PMC7934682; doi:10.7717/peerj.10777)
Supplement: Supplemental Information 4 [file peerj-09-10777-s004.docx]

**Supplement Table 1.** Information of participants recruited from three medical centers

| Cancer Type | Patients from Medical Center | | | Total Patients Number | Gender | | Average Age (years) |
| --- | --- | --- | --- | --- | --- | --- | --- |
|  | SRR | SAH | ZCH |  | Male | Female |  |
| LC | 18 | 62 | 60 | 140 | 82 | 58 | 58.2 |
| CRC | 10 | 31 | 44 | 85 | 50 | 35 | 59.8 |
| BCa | 40 | 25 | 27 | 92 | 0 | 92 | 56.6 |
| GC | 6 | 19 | 45 | 70 | 34 | 36 | 60.4 |
| PDAC | 2 | 58 | 6 | 66 | 39 | 27 | 55.9 |
| CC | 42 | 4 | 4 | 50 | 0 | 50 | 48.4 |
| HNSCC | 4 | 25 | 25 | 54 | 24 | 30 | 50.4 |
| EC | 2 | 10 | 19 | 31 | 14 | 17 | 65.3 |
| BC | 0 | 2 | 19 | 21 | 10 | 11 | 45.1 |
| HCC | 1 | 4 | 14 | 19 | 10 | 9 | 61.3 |
| PCa | 3 | 15 | 7 | 25 | 17 | 8 | 57.6 |
| OC | 12 | 1 | 1 | 14 | 0 | 14 | 57.8 |
| UC | 0 | 0 | 13 | 13 | 6 | 7 | 67.2 |
| Sacroma | 7 | 3 | 0 | 10 | 3 | 7 | 50.4 |
| Healthy | 50 | 11 | 11 | 72 | 36 | 36 | 54.2 |
| Total | 197 | 270 | 295 | 762 | 325 | 437 | 57 |

SRR = Zhejiang University Medical College Affiliated Sir Run Run Shaw Hospital, SAH = Zhejiang University Medical College Second Affiliated Hospital, ZCH = Zhejiang Cancer Hospital, LC = lung cancer; CRC = colorectal cancer; BCa = breast cancer; GC = gastric cancer; PDAC = pancreatic ductal adenocarcinoma; CC = cervical cancer; HNSCC = head and neck squamous cell carcinoma; EC = esophageal cancer; BC = brain cancer; HCC = hepatocellular carcinoma; PCa = prostate cancer; OC = ovarian cancer; UC = bladder cancer.
